# Supplementary material for: The interactome of intact mitochondria by cross-linking mass spectrometry provides evidence for coexisting respiratory supercomplexes
Source: Mol Cell Proteomics. 2017 Dec 8;17(2):216–32. doi: 10.1074/mcp.RA117.000470 (PMC5795388; doi:10.1074/mcp.RA117.000470)
Supplement: Supplemental Data [file supp_RA117.000470_133922_0_supp_23978_fzffwf.zip › spectra_annotation/mito_spectra_annotation/297-1-9-1-17-1.pdf]

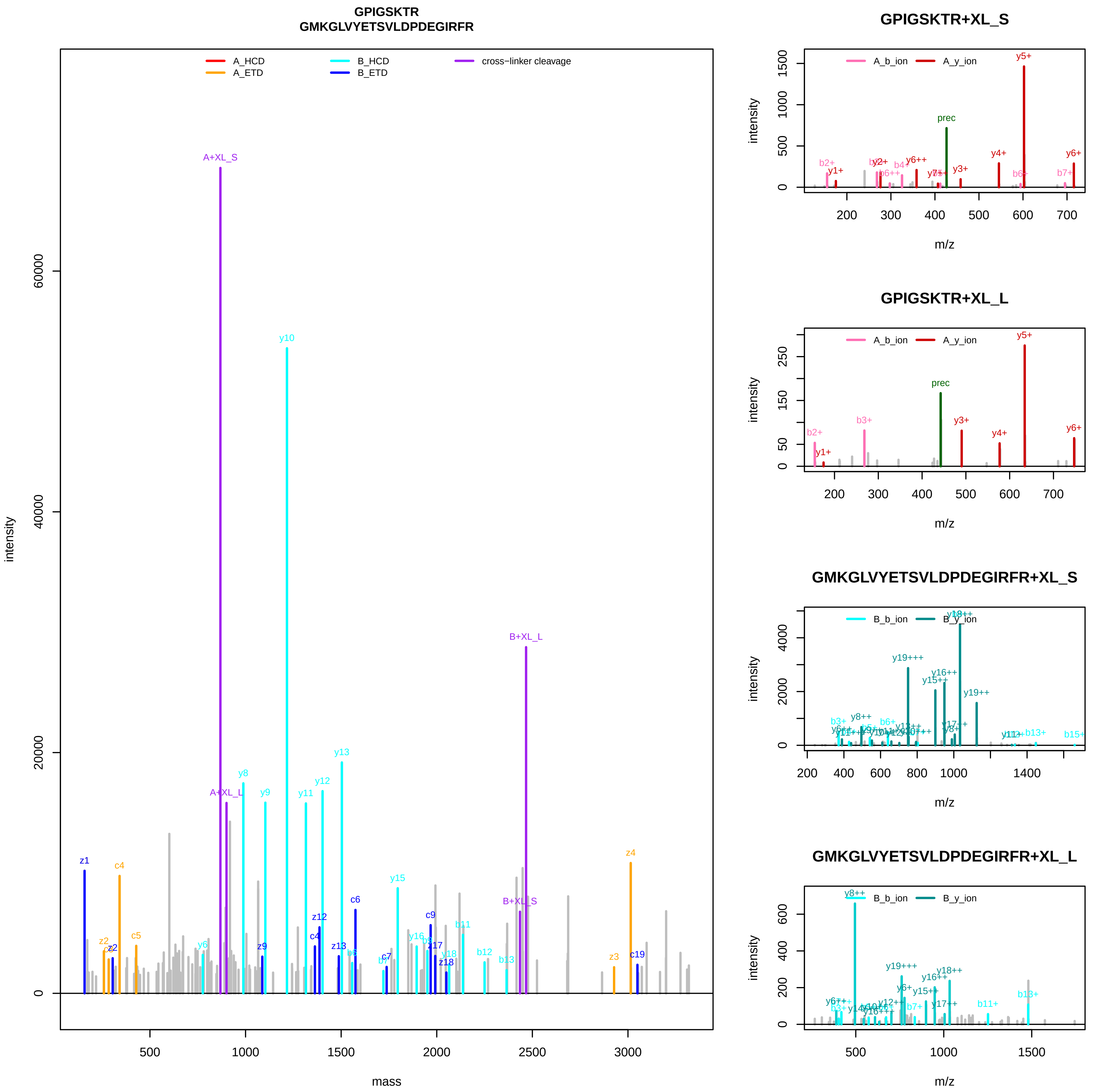
GPIGSKTR
GMKGLVYETSVLDPDEGIRFR
A_HCD
A_ETD
B_HCD
B_ETD
cross−linker cleavage
intensity
mass
A+XL_S
A+XL_L
B+XL_L
B+XL_S
y10
y13
y8
y9
y11
y12
y15
y16
y18
y6
z1
z2
z3
z4
c4
c5
c6
c7
c9
c19
z9
z12
z13
z17
z18
b11
b12
b13
GPIGSKTR+XL_S
GPIGSKTR+XL_L
GMKGLVYETSVLDPDEGIRFR+XL_S
GMKGLVYETSVLDPDEGIRFR+XL_L
A_b_ion
A_y_ion
B_b_ion
B_y_ion
prec
m/z
y5+
y4+
y3+
y6+
y1+
b2+
b3+
y18++
y19+++
y16++
y15++
y19++
b15+
b13+
y8++
b11+
